# Patient Safety in Medical Imaging: a joint paper of the European Society of Radiology (ESR) and the European Federation of Radiographer Societies (EFRS)

**DOI:** 10.1186/s13244-019-0721-y

**Published:** 2019-04-05

**Authors:** European Society of Radiology (ESR), European Federation of Radiographer Societies (EFRS)

**Affiliations:** 1 ESR, Am Gestade 1, 1010 Vienna, Austria; 2 EFRS, Zuidsingel 65, 4331RR Middelburg, The Netherlands

**Keywords:** Education and training, Medical imaging, Patient safety and radiation protection, Radiography, Radiology

## Abstract

The fundamental professional roles of radiographers and radiologists are focused on providing benefit to patients with our skills, while maintaining their safety at all times. There are numerous patient safety issues in radiology which must be considered. These encompass: protection from direct harm arising from the techniques and technologies we use; ensuring physical and psychological well-being of patients while under our care; maintaining the highest possible quality of service provision; and protecting the staff to ensure they can deliver safe services. This paper summarises the key categories of safety issues in the provision of radiology services, from the joint perspectives of radiographers and radiologists, and provides references for further reading in all major relevant areas.

This is a joint statement of the European Society of Radiology (ESR) and the European Federation of Radiographer Societies (EFRS), published simultaneously in Insights into Imaging [DOI:10.1186/s13244-019-0721-y] and Radiography (DOI: 10.1016/j.radi.2019.01.009).

## Key points

- While conferring enormous benefits on patients, radiological modalities, techniques, and procedures also involve some risks to the health and well-being of patients;
- Fundamental to all applications of radiological techniques is the requirement that all possible efforts should be made to ensure patients are no worse off after their interaction with radiographers and radiologists than before;
- Safety issues under a variety of headings are considered and explained, ranging from: direct results of exposure to radiation, through drug and contrast use, to less-obvious topics such as data protection and communication issues;
- As part of a team caring for patients, radiologists and radiographers have responsibility for patient safety; joint attention to the primacy of patient safety in all we do is key to ensuring a safe environment for our patients.

## Introduction

Since the announcement of their discovery by Röntgen in December 1895, X-rays and the radiological techniques associated with their use have become increasingly central tools in medical diagnosis and management. As a result of the growth in the usefulness of imaging, other, non-radiation-based, imaging techniques have been developed (e.g. ultrasound and magnetic resonance imaging), and image-guided interventional means of treating patients have become commonplace. The benefits to patients from these methods of investigation and treatment have been immeasurable. However, it would be unwise to imagine that no harm can come to patients from the use of radiation-based and other imaging techniques, or from interventional radiology procedures. As with all areas of medicine, certainty of unqualified benefit cannot be provided, and appropriate judgment of the relative benefits and risks must by applied at all times. Radiographers and radiologists are specifically trained users of the imaging modalities involved. Paramount in their training is the optimal use of imaging for the benefit of patients and awareness of potential risks from the use of ionising radiation, including the need to minimise the likelihood of harm from inappropriate or excessive radiation use. Many other aspects of radiographer and radiologist practice encompass elements of awareness of, and care for, patient safety. In this paper, the European Society of Radiology (ESR) and the European Federation of Radiographer Societies (EFRS) jointly attempt to highlight many of the areas of patient safety that form part of normal practice for radiographers and radiologists, and which must always be considered when we use our skills to investigate and treat patients.

## Radiation protection

Radiation protection is a key aspect of maintaining the safety of patients in diagnostic and interventional radiology. The three fundamental principles of radiation protection of patients are justification, optimisation, and the application of doses As Low As Reasonably Achievable (ALARA) (ICRP103) [1]. Under the umbrella of EuroSafe Imaging [2], the ESR and EFRS make strong commitments to all aspects of radiation protection of patients, occupational exposure of staff and the general population. A challenging task for European member states is the implementation of Council Directive 2013/59/Euratom (EU-BSS) [3] requirements in the medical sector into national law. The ESR successfully evaluated the activities in transposition with a European Commission tender project [4, 5].

### Justification

The principal aim of medical exposures is to do as much good as possible with as little harm as possible to the patient. The responsibility for the justification of the use of a particular procedure falls on the relevant medical practitioners (ICRP 103). Justification applies at three levels in the use of radiation in medicine:

- *At level I (the most general level), the proper use of radiation in medicine is accepted as doing more good than harm to patients and society.*
- *At level II, a specified procedure with a specified objective is defined and justified (e.g. chest X-rays for patients showing relevant symptoms, or a group of individuals at risk for a condition that can be detected and treated). The aim of level II of justification is to judge whether the radiological procedure will improve the diagnosis or treatment, or will provide necessary information about the exposed individuals.*
- *At level III, the application of a specific procedure to an individual patient should be justified in advance, taking into account the specific objectives of the exposure and the characteristics of the individual involved.*

To support the referral and justification process of radiological procedures, the EU-BSS requires that in all EU member states referral guidelines for medical imaging, taking into account the radiation doses, are available to the referrers. Referral criteria apply at level II justification for common clinical conditions of patients and imaging procedures. With the ESR iGuide initiative [6], the ESR provides up to date and evidence-based European referral criteria embedded in a clinical decision support tool [5].

### Optimisation and DRLs

The International Commission on Radiological Protection (ICRP) defines optimisation as the process of determining what level of protection and safety makes exposures, and the probability and magnitude of potential exposures, “As Low As Reasonably Achievable” (ALARA), economic and societal factors being taken into account. Optimisation applies to all individual patients after level III justification. This means the type of procedure, the dose, other physical imaging parameters, the use of contrast media, and other drugs must be adapted to the individual specific clinical question. As an example, for CT examinations the use of appropriate scan length, number of scan series, dose modulation, and iterative reconstruction are typical optimisation tools. To support the process of optimisation the EU-BSS requires the establishment, regular review and use of “Dose Reference Levels” (DRLs) for all EU member states; the ESR and EFRS support the European DRLs and have collaborated on several projects in this area. These projects include, for paediatric imaging, the PiDRL project [7, 8] which established age and weight dependent DRLs for common paediatric procedures. Today most DRLs are based on anatomical regions or body parts to be examined but only a few are based on clinical questions. The ongoing EUCLID [9] project will establish clinical DRLs for adults where the dose of a procedure for one anatomical region will be modified depending on the clinical question [5].

### Dose incidents

*Justified and unjustified* too-high exposures of patients undergoing a specific procedure, imaging of a wrong body part, or imaging the wrong patient are rare, but may happen from time to time. The term “dose incidents” is a summary of the definition given by the EU-BSS for unintended and accidental overexposures in Article 4 (99): *"medical exposure that is significantly different from the medical exposure intended for a given purpose”.* In the case of diagnostic and interventional radiology this is related to significant overexposures of individual patients with the risk of deterministic effects (individual approach) or a group of patients with the risk of stochastic effects (collective approach). Radiation protection and patient safety require all efforts to prevent such incidents. If incidents occur, the first step should be in any case a local work-up involving the practitioner, staff members, medical physics experts, and/or radiation protection officer. The referrer and patient (and, if relevant, their carers) should be informed about the incident. The interpretation of incidents may include near misses, where an error was detected before performing the procedure [5].

The EU-BSS require that a report be made to national authorities if an overexposure is classified as significant. The problem is that the EU-BSS leaves the definition of “significant” (Article 63) to the implementing authorities of EU member states. This leads to confusion and a very heterogeneous approach across Europe. From a practical point of view, one would like to have reporting criteria based on physical quantities and units and not semantic criteria like “significant”. Instead, DRLs could be used for this purpose. They should not be applied to individual patients and should not be used as dose limits. DRLs are important tools that could be used to identify procedures with too-high exposures, that should be investigated. DRLs are based on physical parameters including: dose area product (KAP), CT dose index (CTDIvol), dose length product (DLP), entrance air kerma (Ka,r), or average glandular dose (AGD) and could be used with factors of relative overexposures or with derived absolute dose values [5].

In May 2018, a questionnaire on implementation of EU-BSS Art. 63 was sent to all European institutional national member societies of the ESR, including the 28 EU member states. The results revealed the difficulties in finding a harmonised approach, with about 50% of countries having no definition for what “significant” means and no physical reporting criteria. As a result, the ESR plans to publish a white paper giving support to the national societies regarding how to find practical solutions with their regulators. The EFRS has also issued a communication, to all of its national societies and educational institutions, offering guidance on aspects of the EU-BSS [5].

## Drug and contrast issues

### Contrast agents

These include iodinated contrast agents for X-ray based studies (including CT), gadolinium-based contrast agents (BCA) for MRI, and microbubbles for ultrasonography. Each of these agent types has specific safety issues (as summarised in Table 1).

**Table 1:** Agent type and respective safety issues

	Iodinated Agents	Gadolinium BCA	Microbubbles
Hypersensitivity reactions	YES	YES	YES
Nephrotoxicity	YES	NO at clinical doses	NO
Metformin and lactic acidosis	YES	NO	NO
Nephrogenic systemic fibrosis	NO	YES	NO
Brain deposition and other organs	NO	YES	NO
Thyrotoxicosis	YES	NO	NO

### Hypersensitivity reactions – duties of radiographer, radiologist or nurse, depending on circumstances and local practices

Before injection of an agent

- Be prepared (training provided, resuscitation trolley available, emergency phone numbers posted);
- Interrogate the patient for previous reactions, grade, and symptoms

During a reaction:

- Adequately treat the symptoms based on the Ring and Messmer classification (Table 2)

**Table 2:** Symptoms based on the Ring and Messmer classification

Grades	Symptoms
I	Cutaneous Mucosa: erythema, urticaria, angioedema
II	Moderate multivisceral: cutaneous ± hypotension ± tachycardia ± cough, dyspnea ± digestive signs
III	Severe mono- or multivisceral signs: cardiovascular collapse, tachycardia or bradycardia ± arrythmia ± bronchospasm ± digestive signs
IV	Cardiac Arrest

After a reaction:

- Blood sampling for Histamine and Tryptase dosages;
- Consult an allergy specialist for skin testing [10, 11].

It is worth noting that not all reports from patients of previous allergic reactions represent true hypersensitivity. It is important to make reasonable efforts to differentiate patients with true histories of previous reactions from those who have had previous incidents due to other factors, but believed by the patient to represent “allergy”. This differentiation is not always easy, but we should remember that failing to perform an indicated contrast-enhanced study (if true hypersensitivity is not really an issue) may indirectly diminish patient safety.

### Nephrotoxicity of iodinated contrast media

Risk factors include: Age > 70 years, reduced renal function (eGFR < 30 ml/min for intra-venous, 45 for intra-arterial injection), large doses, and multiple contrast injections within 48–72 h. Measurement of renal function should be performed prior to injection in at-risk patients. Hydration of high-risk patients is advisable (for hydration protocols see ESUR guidelines) [11–13].

### Metformin

In patients with eGFR > 30 ml/min, metformin administration may be continued normally. If eGFR < 30 ml/min or iodinated contrast is to be given by an intra-arterial route, metformin should be stopped from the time of injection and resumed 48 h later if renal function has not changed.

### Nephrogenic systemic fibrosis

In the past, nephrogenic systemic fibrosis was mainly seen in patients with severe renal insufficiency or on dialysis after injection of linear Gadolinium chelates (high-risk group); most of these agents have been recently withdrawn from the market in Europe.

### Brain deposition

Due to the detection of regions of signal hyperintensity in the deep brain nuclei after multiple injections of Gadolinium chelates, the European Medicines Agency decided in 2018 to withdraw linear Gadolinium chelates from the market, except for liver specific agents [14]. Evaluation of the risk / benefit ratio of multiple injections should be weighted, especially in children or patients with chronic diseases. Further research is required in this area, and European subspecialty imaging societies are encouraged to publish guidelines for the adequate imaging protocols of such diseases (e.g. Multiple Sclerosis, Crohn’s disease).

### Iodinated contrast induced thyrotoxicosis

Administration of iodinated contrast constitutes an iodine load to the thyroid representing many multiples of the daily recommended iodine intake; this can result in hypersecretion of thyroid hormones, with development of thyrotoxicosis in subsequent weeks occurring rarely, particularly in patients with pre-existing Grave’s disease or multinodular goitre. Where possible, patients with existing thyrotoxicosis should not receive iodinated contrast unless a strong indication for its use is present [15, 16].

### Drug administration

The dose of any type of contrast used should be based on the contrast agent concentration, patient weight, and injection protocols, with a view to balancing optimisation of the quality of the information obtained with minimising the risk of adverse effects.

### Intravenous cannulation

For intravenous injection during CT, an IV cannula should be placed with a Gauge size adapted to the injection flow rate required (mainly 18G). Faster flow rates (and therefore larger cannulae) are generally needed for studies dependent on high-concentration arterial opacification including CT pulmonary arteriography. In the event of inadvertent soft-tissue extravasation of contrast, severe injury is extremely rare, and may include skin ulceration, necrosis or compartment syndrome.

Staff responsibilities include:

- Documenting the extravasation with an X-ray or CT
- Treating the patient: limb elevation, ice packs, monitoring
- Reporting in the medical record and informing the referring physician [17].

### Patient handling

Safe patient handling is defined as any activity requiring force to push, pull, lift, lower, transfer or in some way move or support a person or body part [18]. Patient handling issues have the potential to generate serious problems for both patients and healthcare professionals (HCPs) [19]. The first national policy on Safe Patient Handling was introduced into the United Kingdom in 1992 [20]. Since then, similar pieces of legislation and guidelines have been introduced in many countries around the world [18]. Professional bodies and societies, such as the ESR and EFRS, have also produced reports and guidance on aspects of patient safety [21, 22], but less so on safe patient handling practices. All HCPs have a role in preventing injury and in correct patient handling. Within radiology, this task more commonly falls on radiologists, radiographers, nurses, and support staff. In order to promote optimum patient and staff safety, the following roles and responsibilities should be considered with regards to patient handling.

Radiologists, radiographers, nurses, and support staff should have knowledge and demonstrate the skills necessary to:

- Understand their own roles and responsibilities in relation to safe patient handling;
- Undertake relevant training and updates;
- Have awareness of local patient handling policies;
- Recognise patient handling related risks;
- Commit to introducing precautions to reduce patient handling risks;
- Be able to correctly use and maintain patient handling equipment;
- Follow appropriate systems of work;
- Engage in a multidisciplinary approach to safe patient handling;
- Take reasonable care to ensure that their actions do not put themselves or others at risk;
- Commit to reporting patient handling incidents;
- Commit to reviewing incidents and improving patient handling practices;
- Respect the personal wishes of the patient regarding mobility wherever possible;
- Support patient independence and autonomy.

## Patient information: Informed consent and explanatory information for patients

The ESR Patient Advisory Group (PAG) has stated that all care and communication with and about people must be effective, timely, inclusive, and personalised: ‘nothing about me, without me’ [23]. It is a legal requirement that any patient receiving a medical examination involving ionising radiation is informed in a timely and clear manner of the expected diagnostic or therapeutic benefits of the radiological procedure, as well as the radiation risks.

Seeking patient consent prior to undertaking an examination or treatment is a fundamental ethical and legal requirement [24, 25]. Patients become part of the decision-making process about their care by being provided with clear information to enable their participation and being involved in the actual decisions [24–27]. Provision of timely and appropriate information is also a common courtesy and establishes an appropriate relationship of trust between the patient and the referring doctor and radiology department personnel [23]. To achieve this, the needs and values of the patients and carers must be central to service delivery within each clinical imaging service [28].

For each patient it is essential to ensure the following:

- The patient has the right information to make a decision;
- The information has been presented in a way that the patient can understand;
- The patient has shared in the process of decision making and agrees with the outcome [26].

**Fig. 1:**
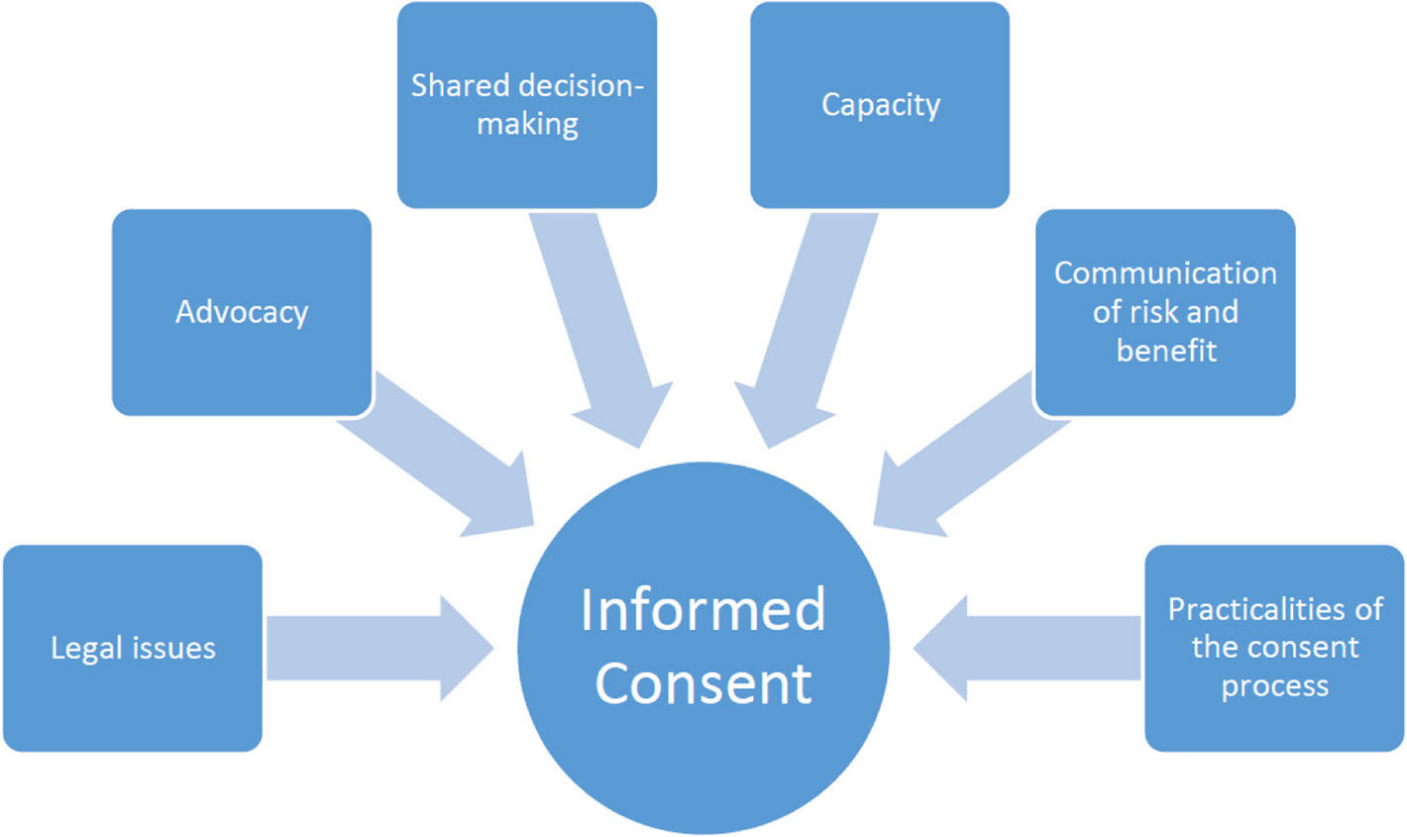
Considerations to take account of when obtaining consent *(reproduced from the Society and College of Radiographers, with their permission)* [24]

There are a number of considerations to take account of when obtaining consent as highlighted in Fig. 1.

- **Legal Issues**
  The patient must be in possession of all the information to make the decision and to be able to do this voluntarily without pressure from external sources.
- **Advocacy**
  It is important that patients have access to the right support to enable them to make their decision. A HCP has the responsibility to identify when a patient may require support and for someone to ‘speak on their behalf’.
- **Shared decision-making**
  The process of consent should be flexible to suit the patient’s needs; it is not a rigid process and time must be allowed for patients to assimilate information prior to being asked to make a decision. The process must be personalised to meet the individual’s needs.
- **Capacity**
  Every person has the right to make a decision and it must be assumed that they have the capacity to do so. Where decisions need to be taken on behalf of an individual this must be done with the interests of the individual being at the centre. It is essential that each professional undertaking the process of obtaining consent is up to date with the national laws in their country and hospital processes with regard to this.
- **Communication of risk and benefit**
  The involved healthcare professionals must inform the individual (or, where appropriate, their carer) of the risks and benefits of the examination and in doing so explain the risks of not having the imaging examination, in a form understandable to the patient [29]. Where possible, patients should be given sufficient time to consider this information fully, before proceeding. Practitioners must respect the patient’s wishes. Many radiological examinations carry a risk. The risks related to radiation exposure and other risks depending upon the examination, for example invasive techniques, should be explained in an understandable form for the patient (e.g. comparative information about the proposed investigation radiation dose in relation to background radiation compared to when flying in an aeroplane). Written information is valuable and can be used by the patient in discussion with their families and carers prior to consenting to the process.
- **Practicalities of the consent process**
  Professionals must follow their hospital and national laws in relation to the practicalities of the consent process. Consent can take verbal and written forms and must take account of paperless systems. Details of the consent process must be fully recorded. Obtaining consent for procedures is a shared responsibility, involving the referring clinician and radiology department personnel; the balance of responsibilities will depend on specific circumstances and working arrangements.

### Other points

- **Children**
  It is important to understand the law in relation to children and consent as this varies across Europe. For example, in the UK, if a child is competent to give consent, the practitioner should take consent from the child. The legal position on competence is different for children under 16 years of age than for children over 16 years.
- **Use of chaperones**
  For intimate examinations it is important to consider the role of chaperones and patients should be able to request a chaperone to support them during the examination. Patients should be made aware that a chaperone can be provided.
- **Consent for research**
  Patients must receive information about the proposed trial and any risks/benefits which may be anticipated. Written information must be provided. Trials involving patients must have ethical approval, as required within the relevant country. Consent in writing is required. Participation in a trial is voluntary and this should be explained clearly to the patient.
- **Consent for education and training**
  Explicit verbal consent must be sought if students/trainees are present for all or part of the imaging examination. Patients must be informed about the number of students/trainees and the role they will play. Patients have the right to refuse care from students/trainees.
- **Emergency imaging**
  There are specific exceptions, where it may not be possible to obtain explicit consent, such as some aspects of emergency care where imaging is required in order to help save the patient’s life or to prevent deterioration of life. If decisions are made in this context, this information should be recorded formally.

In summary, the ESR has published a succinct overview ‘Delivering patient-centred care in Clinical Radiology’ which offers a package of interventions to support delivery of patient-centred care in radiology [23]. Consent to treatment is the principle that a patient must give permission before they receive any type of medical treatment, test or examination. The principle of consent is an important part of medical ethics and international human rights law. For consent to be valid, it must be voluntary and informed, and the person consenting must have the capacity to make the decision. Consent can be given verbally or in writing [30].

## MRI Safety

The major patient safety considerations associated with magnetic resonance imaging (MRI) which must always be considered include:

- The behaviour of ferromagnetic objects when exposed to a strong magnetic field. Forces may act on a ferromagnetic implant causing it to move which could lead to injury and, potentially, death. External ferromagnetic objects may also be influenced by strong magnetic fields and become airborne and move rapidly towards the iscocentre of the magnet. Again this ‘missile’ or ‘projectile’ effect could lead to injury or death.
- Static, or gradient, magnetic fields may also impact on medical devices, implanted or external, and cause these to dysfunction.
- Radiofrequency (RF) related risks include tissue heating due to RF energy deposition which is measured as the specific absorption rate (SAR). This becomes more pronounced with increasing field strengths due to the increased frequency of the RF pulses. RF energy can also be deposited in skin patches, tattoos, cables, and wires, causing them to heat up and, potentially, burn the patient.
- Acoustic noise, associated with the rapidly switching gradient coils, also poses a risk to patients which are avoidable through the appropriate use of hearing protection and noise-reduction technologies.

All professionals involved in MRI i.e. referring clinicians, radiographers, radiologists, and support staff must be aware of these considerations, and appropriate safety procedures must be in place. With over 43 million MRI scans performed across the EU in 2015, together with a 40% growth in MR activity between 2010 and 2015 [31], the need to ensure best practice for patient safety in MRI is vital.

National and international guidance documents related to MR safety are widely available. In 2001, an American College of Radiology (ACR) expert panel produced the *ACR MR Safe Practice Guidelines.* First published in 2002, the latest version, the *ACR Guidance Document on MR Safe Practices: 2013* applies not only to diagnostic settings but also to patient, research subject, and healthcare personnel safety for all MRI settings, including those designed for clinical diagnostic imaging, research, interventional, and intraoperative applications [32]. Safety considerations are discussed under the headings related to: establishing, implementing, and maintaining current MR safety policies and procedures; static magnetic field issues; safety screening; staffing; pregnancy; paediatrics; time varying gradient magnetic field; time varying radiofrequency field; cryogen-related issues; claustrophobia, anxiety, sedation, and anaesthesia; contrast agents; and implants. Similarly, the International Society of Magnetic Resonance in Medicine (ISMRM) have published guidance related to the safe use of MRI for research purposes where they discuss: roles and responsibilities; minimum qualification requirements; operating modes; and considerations for the patient/research subject [33].

A recent consensus document involving eight organisations, including the ESR and the EFRS, made recommendations about clearly defining the roles and responsibilities of those involved in the management of MR safety [34]. They describe the MR Medical Director (MRMD) / MR Research Director (MRRD), the MR Safety Officer (MRSO), and the MR Safety Expert (MRSE); along with the importance of appropriate education and training for such roles.

## Prevention of infection, decontamination, hospital acquired infections

According to the Centers for Disease Control and Prevention (CDC), basic infection control in healthcare facilities should include general principles to avoid transmission in all patient care and also specific ways to prevent transmission in patients that are known or suspected to be infected with a transmittable microorganism [35]. The general principles to contain infection, and to be applied by all healthcare professionals, are based on the following [35]:

- Perform hand hygiene, using the five moments [36] (before touching a patient, before clean/aseptic procedures, after body fluid exposure/risk, after touching a patient, and after touching patient surroundings) and by correctly using an alcohol-based solution for disinfection or by washing hands in an appropriate way;
- Use personal protective equipment whenever an exposure to infectious material might occur;
- Respect and instruct patients on how to sneeze and cough appropriately and in appropriate use of masks in infectious disease;
- Be aware of and ensure staff awareness of issues relating to patients in isolation, namely the respiratory and the contact types of isolation, or both;
- Ensure the appropriate cleanliness of materials (disinfection and sterilisation) and the environment surrounding the patient;
- Handle textiles and laundry garments carefully and dispose of them according to the healthcare facility policies;
- Ensure safe injection procedures, namely the rule “one needle, one syringe and one use”;
- Ensure sharps safety.

Transmission prevention procedures to be used in patients suspected, or confirmed, of being infected or colonised with certain infectious agents should include the following [35]:

- Contact precaution to be used when contact transmission might occur. This could be achieved by isolation of the patient, use of personal protective equipment, limiting transport and movement, use of disposable equipment when possible, and ensuring cleaning of surfaces;
- Droplet precaution to be used in case of infectious agents that might be transmitted by respiratory droplets, when a patient is sneezing, coughing, or talking. This could be achieved by continuous use of patient facemasks, ensuring appropriate patient placement, use of personal protective equipment and limiting patient movement and transport;
- Airborne precautions when infectious microorganisms have an airborne route, such as tuberculosis, measles, or chickenpox. This could be achieved by respecting droplet precaution plus the restriction of susceptible healthcare professionals entering the room and by immunisation of those susceptible persons.

The radiology department has usually been considered a low risk environment for infections associated with healthcare (nosocomial infections), but the potential for transmission of infectious pathogens to both patients and healthcare professionals exists [37]. Radiology departments have a steady stream of a wide variety of patients each day. Patients referred from ambulatory care and emergency departments often mix with inpatients. All of these patients can contaminate the environment of the radiology department with pathogens. Given the large number of patients with confirmed infections and those undiagnosed as infected that go to the radiology department, and the potential to contaminate both objects and the air with pathogens, surface cleaning must be done between all patients, with more rigorous cleaning protocols at periodic intervals. The radiology department must also maintain good communication with the clinical areas referring patients for radiologic procedures, in order to properly identify those patients that need extra precautions [38].

In the specific case of the radiology department, chin supports and chest racks used to obtain chest radiographs, anatomical markers, fluoroscopy equipment, X-ray tubes, and X-ray receivers may all become contaminated with multiple microorganisms from patients, with the potential for spread to other patients, if proper measures are not taken [38].

### Ultrasound infection prevention

Most non-invasive radiological procedures (e.g. radiography and CT) do not involve direct contact of equipment with potentially-infected surfaces, given the usual presence of intervening clothing and/or dressings. Ultrasound is an exception to this. Because adequate ultrasound imaging requires good transducer-to-skin contact (or endocavitary or other intracorporeal positioning of a transducer), the potential exists for transmission of infection between patients via transducers [39, 40]. Ultrasound gel is another potential means for infection transmission, especially if multi-use gel dispensers are utilised [39, 40]. It has been shown that bacterial contamination of ultrasound transducers is significantly higher than that of bus poles and public toilet seats [40, 41].

Survival times for some viruses, bacteria and fungi on dry inert surfaces (including transducer surfaces) can be up to several months, or even longer if the surface is contaminated with co-existent organic material [40]. A recent survey of European practices regarding ultrasound probe cleaning and decontamination, probe cover and sterile gel use found wide variation among respondents, with no uniformity of approach [39].

Accordingly, the Ultrasound Working Group of ESR published a set of recommendations in 2017 aimed at providing ultrasound users with a set of standards for ultrasound transducer decontamination, use of transducer covers and gel [40]. These are aimed at minimising the potential risks to patients from ultrasound studies and procedures. The recommendations cover equipment and gel contact with intact surface skin, mucous membranes, bodily fluids (including interventional procedures), and infected/broken skin and wounds, including protocols for cleaning and disinfection of transducers after every examination in each circumstance.

## Data security and new IT developments

Radiologists and radiographers have been at the forefront of adopting digital medical imaging and electronic health information. Radiologic images, lab test results, medications and other clinical information are now typically stored and viewed on computers. The responsibility that physicians have to protect their patients from harm extends to protecting patient information, privacy, and confidentiality.

To provide high quality medical care to patients, radiologists and radiographers use information from the Hospital Information System (HIS), Radiology Information System (RIS), and Picture Archiving and Communication System (PACS). The ESR endorses the view that the radiologist who interprets images of a patient for diagnostic purposes or who performs interventional image-based procedures should have full access privileges as a consultant to all medical data including all previous images, as well as clinical, chemical, and biological analyses [42].

Work with this electronic medical information has to be performed within a safe and secure environment. It is the responsibility of all health care professionals in a radiology department to ensure that electronic medical information is properly protected. Therefore, radiology organisations have to make sure that policies and standards related to the protection of medical information are in place. Access to databases such as Electronic Patient Record (EPR) and PACS is currently mainly regulated by local rules, created by the hospital administration, or by a national authority.

Applied across the European Union (EU) since 25 May 2018, the new General Data Protection Regulation (GDPR) addresses the protection of EU residents with regard to accessing, processing and the free movement of their personal data [43, 44]. The regulation aims at protecting the confidentiality of personal health data whilst preserving the benefits of digital image processing for research and public health purposes. The new GDPR makes ‘data protection by design and by default’ an essential principle. Radiology departments specifically have to:

- Obtain explicit consent from the data subject (the patient) prior to processing or communicating his or her data, unless in situations where derogations exist;
- Apply appropriate technical and organisational safeguards such as anonymisation, pseudonymisation, and encryption for data use in the context of public health projects, individual research projects, or imaging biobanks for ‘big data’ analysis;
- Provide access for the data subject to his/her personal medical records containing information such as diagnoses, examination results, assessments by treating physicians, and any treatment or interventions provided.

Artificial Intelligence (AI) and Deep Learning (DL) technologies are growing in importance in the radiology sphere. These involve innovative ways of using imaging (and other) data to enhance the diagnostic process, and may have profound impacts on the practice of radiology in the future. AI and DL in radiology require training of algorithms on large annotated datasets, which raises further issues of data protection and consent that will have to be addressed comprehensively [44]. Although anonymised data sets are not subject to the GDPR, it is difficult to define exactly the conditions which should be fulfilled in order to reliably anonymise digital image data for research and development purposes. For example, ethical issues include the potential for reversal of de-identification or anonymisation of patient data through data-linking DICOM tags or face-recognition software, the need to ensure equality of access and absence of bias in algorithms, and the lack of clarity about intellectual property rights that could arise from use of patient data to develop and market potentially highly-profitable AI products [45]. Many radiology societies, including the ESR, have published or are developing position papers explaining these novel developments and the associated issues to the radiology and patient communities.

## Appropriate professionals

In most developed countries, the range of tasks involved in the performance and interpretation of imaging studies are performed by respectively, radiographers and radiologists **(**usually collaboratively, with each profession responsible for specific elements of the process) who have completed formal training programmes conforming to national and international standards, following approved curricula, and requiring accumulation of defined minimum amounts of experience in their profession and specialty. This is entirely appropriate, and ensures that patients have access to safe, optimised services.

The benefits to patients from this include:

- Radiation protection (see earlier section): Dose optimisation and radiation protection are key components of the training undergone by qualified radiographers and radiologists.
- Appropriateness of investigations: Properly-trained and -qualified radiologists are best-placed to judge whether a requested study or procedure represents the best method of obtaining the information required or achieving the result desired. In many instances, alternative studies may be safer and more helpful, or studies requested may not be appropriate to answer the clinical question. Untrained or incompletely-trained individuals lack the breadth of knowledge and understanding required to always choose the wisest and safest method of investigation, and may be more likely to follow pathways of investigation that fit with their particular interests, knowledge or preconceptions (“if you only have a hammer, everything looks like a nail”).
- Clinical Decision Support (CDS): CDS software packages have been developed by a number of major radiology societies, including the ESR, with the intention of providing referring clinicians (and radiology professionals) with guidance regarding appropriate radiological investigative pathways [46]. The use of CDS depends crucially on the specific knowledge and experience of trained professional radiographers and radiologists.
- Subspecialisation: this has particular application in interpretation of imaging studies. Access to subspecialty-trained radiologists ensures optimal information retrieval from investigations. This cannot be guaranteed when interpretation is performed by medical or non-medical individuals who lack the training and experience of specialists and subspecialists.

In many instances, non-radiologist interpretation of images is performed by other medical specialists, focusing on their particular interest or question. This is understandable, but not optimal, and this focused interpretation runs the risk (among others) of not identifying or recognising unexpected abnormalities outside the area of their particular focus. As a minimum standard, if such non-specialist interpretation is performed, a formal report should be generated and recorded accessibly by the interpreter [47], available afterwards for review.

In the context of the growing emphasis on Value-Based Healthcare models, substantial value is provided to the patient by having their imaging and interventional radiological procedures performed and interpreted by trained professionals [48], and there should be no room in any developed society for uncontrolled, unregulated, “amateur” performance and interpretation of studies.

## Interventional radiology

Interventional radiology (IR) procedures are subject to all the risks to patient safety that apply to any radiological procedure, and all appropriate precautions taken in the setting of other modalities must also be observed in IR. IR procedures also carry additional risks to patients, relating to potential complications or negative outcomes of the relevant procedures, and the possibility of injury or harm resulting from insufficient care being applied before and during procedures. Drug use during IR procedures may include (among others) sedatives, vaso-active substances, analgesics, and antibiotics; each of these can have specific safety issues.

Surgical checklists have become commonplace tools to reduce morbidity and mortality in surgery. This concept has more recently been applied to IR [49, 50], with the development of pre-procedural, sign-in and sign-out templates for IR procedures, designed to ensure that the correct procedure is being performed on the correct patient, that all relevant information is available, that all appropriate safety steps are observed, and that clear post-procedural planning is put in place and communicated.

## Protection of children and other vulnerable persons

Aside from examination or procedure-specific paediatric considerations, child protection is an extremely important issue in patient safety. According to the European Commission [51] and UNICEF [52], comprehensive national child protection systems must be in place which should apply to all aspects of a child’s life. There is an onus on all health professionals in medical imaging departments to respect and protect the rights of the child. In many jurisdictions, radiologists and radiographers will also have legal responsibilities related to the reporting of suspected physical abuse of children/suspected non-accidental injury. While some children may be referred for imaging examinations where there is a pre-existing suspicion of abuse, other cases may only become apparent when radiologists, and radiographers in particular, are interacting with children and their parents or guardians.

Attention must also be given to other vulnerable persons which may include: the elderly, those with memory complaints, intellectual disabilities, or mental health issues. In any such cases, appropriate systems to facilitate communication and consent should be in place. These systems must include the requirements for responsible adults, guardians, or chaperones.

## Communication

### Communication between patients and radiology staff

Patients, during an occurrence of disease or phase of care, can come in contact with a broad variety of different healthcare professionals, each an important link of the healthcare chain.

The radiology department has a constant stream of a wide variety of patients each day, referred from ambulatory care and from the emergency department and inpatients. All of these patients are likely to have contact with radiology professionals, and possibly with other members of the radiology multidisciplinary team. At each step and for each professional, good communication skills are vital. In order to involve a patient in his/her own healthcare process, it is of maximum importance to explain the whole examination or procedure in a structured way. To do so, the following topics should be taken into account and, whenever possible, respected [53]:

- Use of verbal and non-verbal communication to let the patient feel at ease;
- Asking the patient their opinions and thoughts to allow shared management decisions;
- Recognising and acknowledging their emotions and fears and allowing them time to express them;
- Avoiding (where possible) the use of complicated medical and technical terminology and checking understanding all the way through the explanation;
- Allowing the patient or relative (or the legal guardian) time to ask questions and offering follow-up if needed;
- Respecting the need for autonomy of the patient, even if their viewpoint may be different from the professional’s understanding.

It is important to make sure that the correct patient is about to undergo a given radiologic procedure or examination. Examining the wrong patient could lead to unnecessary exposure to ionising radiation, to misdiagnosis of a severe pathology, or even to an unnecessary intervention.

In order to avoid this, the following should be done [54]:

- ASK the patient to state (and where possible/practical spell) their full name and date of birth;
- ALWAYS check this against the patient identification band, which must say exactly the same;
- NEVER ask the patient “*are you Mr Jones*?”; the patient may have misheard and mistakenly agree;
- NEVER assume the patient is in the right bed or that the name tag above the bed is correct.

Again, whenever possible, after the patient enters the examination room, and immediately before any diagnostic or therapeutic action or procedure, with the patient present, verbally (and/or in the patient identification bracelet) confirm [55]:

- Correct patient is present;
- Correct examination is about to be performed;
- Clinical history corresponds to the requested examination;
- Correct side or site is being examined;
- Right or left side markers, acquisition of the topogram, the ultrasound transducer, etc. are being used correctly and according to the side/extremity.

### Communication among professionals (radiographers, radiologists, referrers/including handover)

Critical information must be accurately communicated between radiology department staff and staff from other departments or, within the radiology department, between professionals. For example, proper identification of those patients that need extra precautions (protective or contact isolation), are at a higher risk, or cannot wait for their turn in the waiting room depends on these communications channels. In a hospital, patients often move between areas of diagnosis, treatment, and care on a constant basis and may even come across several shifts of staff each day, which introduces an additional safety risk to the patient each time change happens [56]. In all instances, the information communicated about a patient and the handover of a patient or a shift must achieve a balance between comprehensiveness and efficiency [55].

It is crucial to keep in mind that the information provided during handovers of patients or shifts will influence the delivery of care for the whole shift and important or critical information can be lost, leading to gaps in patient care [57]. The use of the SBAR (Situation, Background, Assessment and Recommendation) tool, with the due adaptations for communicating critical information, is recommended [58]:

- **S**ituation – What is going on with the patient or the situation? For an emergency patient, ask what the presenting complaint is. For an inpatient, ask what the current concern is;
- **B**ackground – What is the clinical background or context? What is the relevant past medical story or what has happened during this inpatient admission?
- **A**ssessment – What do I think the problem is? Ask for the current set of observations and relevant clinical findings;
- **R**ecommendation – What would I do to correct it? What needs to be done now? Are there any outstanding jobs? How urgent is it?

The professional handing over the shift or the patient may use the 4 ‘R’ technique [59]:

- **R**elevant items that will be items that will be **R**emembered (focus on sickest patients first; daily progress and direction on what to do);
- Give directions with **R**ationale avoiding ambiguity;
- Check for **R**eceiver understanding, encouraging questions.

On the other hand, the individual who is receiving the information should:

- Listen actively (being focused on receiving information, limiting interruptions during the handoff, and taking notes if needed);
- Ask questions (to ensure the understanding of what is being said);
- Use a system (to keep track of to-do items that require action);
- Read back (directions to ensure you are on the same page).

In addition to the shift or patient handover procedure, it is also very important to [55]:

- Assign enough time for communicating important information and for staff to ask and respond to questions without interruptions wherever possible (repeat-back and read-back steps should be included);
- Provide information regarding status, medications, treatment plans, advance directives, or any significant status changes;
- Limit the exchange of information to that which is necessary to provide safe care to the patient.

## Quality improvement

*“Well, nobody’s perfect”* [60].

No radiographer, radiologist, or radiology department is perfect, and quality improvement is always possible. This should be embraced and utilised to become embedded in daily practice, such that any and all opportunities for reflection on performance, outcomes and interactions with patients are used to influence learning and to initiate change, where needed.

1. **Errors** are part of all human activity. Every effort should be made to avoid error, but when it happens, it should be acknowledged (and shared with the patient if that would benefit patient care). A no-blame culture should be encouraged within departments, such that errors are used as learning opportunities for all, not as tools to isolate or demean individuals. One possible mechanism for such learning is through open review of errors by all relevant department members, to identify possible causes and methods of eliminating those causes in the future (e.g. learning from discrepancy meetings) [61, 62].
2. **Continuing professional development (CPD).** We work in rapidly-changing disciplines; this brings both excitement and challenge to our work. We cannot assume that it is appropriate to practise for a lifetime on the basis of what we knew when we completed formal training. Continuous education is necessary for us to serve our patients properly, adapting what we do to new technologies, developments, and changing circumstances. All radiographers and radiologists should incorporate a culture of continuous learning into their practice, and should be supported in doing so by their professional and national societies (by provision of timely and up-to-date educational opportunities) and by their employers (by provision of protected time and resources). Personal learning and intra-departmental CPD should be encouraged, and, ideally, provided for in work schedules [63].
3. **Peer review.** Many opportunities for peer review of our work are already part of daily activity in radiology departments: review of prior study reports when reporting a new study, review of the quality of imaging when reporting a study, review of multiple studies and change over time when conducting multi-disciplinary team conferences, review of studies from outside institutions imported for specialist or subspecialist opinions, etc. All of these offer opportunities for us to assess the quality of work of our peers, and of ourselves. These are all valuable opportunities for learning, and for two-way communication to optimise the work output of the person whose work is being reviewed and of the reviewer. As with errors and discrepancies (item 1), this should always take place in a blame-free environment focused only on quality improvement for the future [64].
4. **Clinical Audit.** European Union directives have mandated the performance of clinical audit in radiology departments since 1997. This requirement is emphasised in the current EU-BSS [3, 65]. Clinical audit is a simple yet powerful tool for evaluation of current practices, and for providing guidance to change and improve those practices when appropriate. Essentially, clinical audit involves measuring what we do against a standard, and then changing what we do to allow us meet that standard when appropriate. The ESR has developed a booklet explaining clinical audit and providing a series of templates for performance of audit to assist departments beginning this activity [22, 66].
5. **External review.** In some countries, external reviews of radiology departments will be a legal requirement, assessing their activities against standards. While such inspections may be stressful for departments and the professionals who work within them, they can also provide an impetus to optimise performance and safety standards.
6. **Risk management.** Whatever the legislative arrangements in individual countries, the process of managing and minimising risk within radiology departments should be primarily the responsibility of the professionals working within the department (assuming adequate resources are made available). Group responsibility and peer evaluation are fundamental to effective risk management; the members of the radiological team should be the principal actors in maintaining quality within their own organisations. Clinical audit is an essential component of this activity. Continuing quality improvement activities are a major component of promoting competence within radiological teams.

This is not intended to be an exhaustive list of quality improvement activities in which professionals in radiology can engage; rather, it is intended to provide examples of areas where a continuous culture of attention to quality improvement can be incorporated into normal professional work. The focus of quality improvement activities will vary from country to country and from department to department. Whatever the local needs and indicators, each radiology department should devote regular attention to assessing the quality of its work, and, where possible, seeking to continually improve it.

## Fatigue / Burnout

Burnout is a state of mental weariness which has been initially defined as a sustained response to chronic emotional and interpersonal stressors within the workplace [67]. Reports also describe burnout as the progressive loss of energy and enthusiasm [68]. Burnout has been shown to lead to decreases in productivity and effectiveness, reduced commitment to the job, and negative effects on home life [69]. Causes of burnout are multifactorial but having too many bureaucratic tasks, too many work hours, and increasing levels of computerisation have been linked. Within the radiology literature, reports regarding burnout within clinical practice are increasing in frequency [67, 70, 71].

Similar reports exist with regard to the issue of fatigue. Waite et al. [72], in 2017 compiled a review on the influence of fatigue within radiology. In this paper, fatigue was similarly defined as a weariness and depletion of energy that can manifest physically and cognitively. Both burnout and fatigue have huge implications in terms of their effects on patients, colleagues, and the individuals concerned. Visual and mental fatigue among radiology professionals have been shown to occur towards the end of long work-days, and to have negative effects on lesion detection and decision-making [61].

The American College of Radiology (ACR) Commission on Human Resources recommends [73] that radiology leaders and departments consider the following actions to help mitigate the risk of burnout and fatigue:

- **Have adequate staffing**

Ensure that suitable staffing levels appropriate to the workload are maintained.

- **Reduce prolonged stress**

Ensure appropriate scheduling of duties, time off for rest, a reasonable pace of work, and fairness in the workplace.

- **Restore a sense of control**

Emphasise the importance of teamwork, involve radiology staff in decision making, recognise good work, encourage respectful and compassionate treatment of all. Develop high quality effective communication skills. Improve or resolve problems within departments. Review job satisfaction as part of a regular personal development review process.

- **Restore lifestyle balance**

Support colleagues in solving problems with lifestyle balance which may include physical, emotional, spiritual, and relationship-related aspects.

- **Reduce out-of-hours obligations**

Consider recruiting staff members who would prefer to work on out-of-hours shifts. Shorter shifts may also be useful for high intensity roles.

- **Improve staff efficiency**

Optimise the use of support staff. Develop greater efficiencies in workflow. Increase the connectivity and functionality of PACS and other related IT systems.

- **Reduce isolation**

Encourage staff to work as a part of teams. Improve communication between radiology colleagues and those outside of radiology. Encourage staff to take breaks in common areas i.e. staff rooms

- **Develop reasonable expectations and goals**

Set expectations and goals based on the volume of work and the availability of staff. Monitor work quality, turnaround times, patient and referrer satisfaction.

- **Provide professional help**

Consider providing workplace interventions designed to prevent or treat burnout/fatigue. Consider implementing interventions designed to change how an organisation manages burnout/fatigue.

- **Promote action by the Radiology Community**

Encourage the Radiology Community (radiologists, radiographers, and support staff) to have greater awareness of burnout/fatigue and implement practices to mitigate the problem moving forward. Professional societies, such as EFRS and ESR, should continue to raise awareness and propose solutions to burnout and fatigue.

## Training in patient safety issues

The Statutes of the ESR [74] and Constitution of the EFRS [75] clearly highlight the importance placed on education and training by both organisations. There has been much discussion on the potential for greater focus on patient safety, including within undergraduate curricula, to effect change [76–80]. Embedding patient safety in curricula can help us move toward a patient safety culture and a system of safety but this requires careful consideration of the learning system [81]. Educational activity related to patient safety must be transparent and consistent within curricula, it must be a core theme throughout, and it must be regularly reviewed and enhanced; thus, requiring a holistic approach.

To aid education and training providers, the ESR has published three European Training Curricula for Radiology: *Curriculum for Undergraduate Radiological Education* [82], *European Training Curriculum for Radiology (Level I and II)* [83], and *European Training Curriculum for Subspecialisation in Radiology (Level III)* [84]. All three clearly indicate the importance of patient safety with defined learning outcomes and topics. Similarly, the EFRS has published their *European Qualifications Framework (EQF) Level 6 (Bachelors) Benchmarking Document for Radiographers* [25] and *EQF Level 7 (Masters) Benchmarking Document* [85] which also highlight the intricate nature of patient safety-related content. Additionally, the ESR led the EC-MEDRAPET project (also involving the EFRS) that established European guidelines on radiation protection education and training of medical professionals in the EU [86]. The ESR and EFRS curricula and benchmarking publications provide a framework to facilitate the mapping of activity, yet despite the growing focus on patient safety, there is a paucity of published reports exploring the inclusion of patient safety topics within radiology and radiography curricula. The EFRS thus undertook a project to evaluate and report on the inclusion, and assessment, of patient safety-related topics within undergraduate radiography curricula across Europe [21]. This study, which surveyed 33 educational institutions across Europe, revealed that while most patient safety topics appeared to be taught across most programmes, several important topics were only taught at an introductory level in some centres. Variability was also apparent in terms of the teaching and assessment methods used. While the findings of this study were reassuring, opportunities to further advance patient safety education and training within curricula were identified, and both the ESR and the EFRS have a key role to play through continued promotion.

## Conclusion

A simplistic view of patient safety in radiology is that the key risk relates to inappropriate radiation exposure. While preventing this is a central part of the responsibility of radiographers and radiologists, there is a much wider range of patient safety aspects of the work of radiology professionals. In this paper, we have not attempted to provide a comprehensive list of all safety issues. Our focus, rather, has been to highlight certain broad headings to provide a resource for those radiographers and radiologists who wish to find relevant guidance and references. In addition, the ESR and EFRS seek to keep safety considerations central to future educational, resourcing and development planning in patient care, as it applies to our specialties and our patients. This joint paper, reflecting the concerns and understanding of the European radiographer and radiologist communities, is a key component of explaining and highlighting the range and complexities of our duties and responsibilities to ensure the best possible outcomes for our patients. Local practices will determine to some extent how these safety standards are implemented in each country, but the fundamentals of our work are the same everywhere: our patients are central to our work, and their safety must always be paramount.
